# *EIF3F*-related neurodevelopmental disorder: refining the phenotypic and expanding the molecular spectrum

**DOI:** 10.1186/s13023-021-01744-1

**Published:** 2021-03-18

**Authors:** Ulrike Hüffmeier, Cornelia Kraus, Miriam S. Reuter, Steffen Uebe, Mary-Alice Abbott, Syed A. Ahmed, Kristyn L. Rawson, Eileen Barr, Hong Li, Ange-Line Bruel, Laurence Faivre, Frédéric Tran Mau-Them, Christina Botti, Susan Brooks, Kaitlyn Burns, D. Isum Ward, Marina Dutra-Clarke, Julian A. Martinez-Agosto, Hane Lee, Stanley F. Nelson, UCLA California Center for Rare Disease, Pia Zacher, Rami Abou Jamra, Chiara Klöckner, Julie McGaughran, Jürgen Kohlhase, Sarah Schuhmann, Ellen Moran, John Pappas, Annick Raas-Rothschild, Maria J. Guillen Sacoto, Lindsay B. Henderson, Timothy Blake Palculict, Sureni V. Mullegama, Houda Zghal Elloumi, Adi Reich, Samantha A. Schrier Vergano, Erica Wahl, André Reis, Christiane Zweier

**Affiliations:** 1 Institute of Human Genetics, Universitätsklinikum Erlangen, Friedrich-Alexander-Universität Erlangen-Nürnberg (FAU), Schwabachanlage 10, 91054 Erlangen, Germany; 2 Medical Genetics, Department of Pediatrics, University of Massachusetts Medical School – Baystate, Springfield, MA USA; 3 Department of Genetics, Southern California Permanente Medical Group, Kaiser Permanente, Riverside, CA USA; 4 Department of Human Genetics, Emory University School of Medicine, Atlanta, GA 30322 USA; 5 UMR-Inserm 1231 GAD Team, Génétique des Anomalies du développement, Université de Bourgogne Franche-Comté, 21000 Dijon, France; 6 Laboratoire de Génétique Chromosomique et Moléculaire, UF Innovation en diagnostic génomique des maladies rares, Plateau de Biologie Hospitalo-Universitaire, Centre Hospitalier Universitaire de Dijon, Dijon, France; 7 Centre de Génétique, Centre de Référence «Anomalies du Développement et Syndromes Malformatifs» et FHU TRANSLAD, Hôpital D’Enfants, Centre Hospitalier Universitaire de Dijon, Dijon, France; 8 Division of Medical Genetics, Department of Pediatrics, Rutgers Robert Wood Johnson Medical School, New Brunswick, NJ 08901 USA; 9 Sanford Health, Sioux Falls, SD USA; 10 Division of Genetics, Department of Pediatrics, David Geffen School of Medicine, University of California at Los Angeles, Los Angeles, CA 90095 USA; 11 Department of Human Genetics, David Geffen School of Medicine, University of California at Los Angeles, Los Angeles, CA 90095 USA; 12 Pathology and Laboratory Medicine, David Geffen School of Medicine, University of California at Los Angeles, Los Angeles, CA 90095 USA; 13 UCLA California Center for Rare Diseases, David Geffen School of Medicine, University of California at Los Angeles, Los Angeles, CA 90095 USA; 14 Institute of Human Genetics, University of Leipzig Medical Center, Leipzig, Germany; 15 Epilepsy Center Kleinwachau, Radeberg, Germany; 16 Genetic Health Queensland, Royal Brisbane and Woman’s Hospital, Brisbane, Australia; 17 School of Medicine, The University of Queensland, St Lucia, Brisbane, Australia; 18 Synlab Human Genetics Freiburg, Freiburg, Germany; 19 Clinical Genetics, Hassenfeld Children’s Hospital at NYU Langone, NYU Langone, Orthopedic Hospital, New York, NY USA; 20 Division of Clinical Genetic Services, Department of Pediatrics, NYU Grossman School of Medicine, New York, NY USA; 21 Sackler School of Medicine at Tel Aviv University, Tel Aviv, Israel; 22 Institute of Rare Diseases, Edmond & Lily Safra Children Hospital, Tel Hashomer, Israel; 23 GeneDx, Gaithersburg, MD 20877 USA; 24 Division of Medical Genetics and Metabolism, Children’s Hospital of The King’s Daughters, Norfolk, VA USA; 25 Division of Genetics, UBMD Pediatrics, Buffalo, NY USA; 26 Present Address: Department of Human Genetics, Inselspital, Bern University Hospital, University of Bern, Bern, Switzerland

**Keywords:** *EIF3F* gene, Neurodevelopmental disorder, Short stature, Deafness, Behavioral difficulties, Altered muscular tone

## Abstract

**Background:**

An identical homozygous missense variant in *EIF3F*, identified through a large-scale genome-wide sequencing approach, was reported as causative in nine individuals with a neurodevelopmental disorder, characterized by variable intellectual disability, epilepsy, behavioral problems and sensorineural hearing-loss. To refine the phenotypic and molecular spectrum of *EIF3F-*related neurodevelopmental disorder, we examined independent patients.

**Results:**

21 patients were homozygous and one compound heterozygous for c.694T>G/p.(Phe232Val) in *EIF3F*. Haplotype analyses in 15 families suggested that c.694T>G/p.(Phe232Val) was a founder variant. All affected individuals had developmental delays including delayed speech development. About half of the affected individuals had behavioral problems, altered muscular tone, hearing loss, and short stature. Moreover, this study suggests that microcephaly, reduced sensitivity to pain, cleft lip/palate, gastrointestinal symptoms and ophthalmological symptoms are part of the phenotypic spectrum. Minor dysmorphic features were observed, although neither the individuals’ facial nor general appearance were obviously distinctive. Symptoms in the compound heterozygous individual with an additional truncating variant were at the severe end of the spectrum in regard to motor milestones, speech delay, organic problems and pre- and postnatal growth of body and head, suggesting some genotype–phenotype correlation.

**Conclusions:**

Our study refines the phenotypic and expands the molecular spectrum of *EIF3F*-related syndromic neurodevelopmental disorder.

**Supplementary Information:**

The online version contains supplementary material available at 10.1186/s13023-021-01744-1.

## Introduction

Bi-allelic variants in the *EIF3F* gene have recently been published as the cause for a syndromic neurodevelopmental disorder (NDD) (OMIM #618,295: intellectual developmental disorder, autosomal recessive 67). Variants were identified by a large exome-wide recessive burden analysis of > 4500 families with no previous molecular diagnosis [9]. All nine affected individuals from seven families carried the same homozygous *EIF3F* missense variant c.694T>G/p.(Phe232Val). Beside variable intellectual disability (ID) in all individuals, epilepsy occurred in six, and behavioral problems or sensorineural hearing loss in three individuals, respectively [9]. Rarer malformations described in single individuals were bilateral cleft lip and palate, congenital lobar emphysema, anal stenosis and undescended testis. Neurological rarer symptoms included a high pain threshold, drop attacks, muscular hypoplasia; upon brain imaging, an arachnoid cyst in the right cerebellar pontine angle, prominent perivascular spaces and mild tonsillar ectopia were observed in single patients.

*EIF3F* encodes an essential subunit of the largest eukaryotic translation initiation factor eIF3 which binds to a highly specific group of mRNAs involved in cell proliferation and growth, including cell cycle control, differentiation and apoptosis [8, 10]. In vitro studies of induced pluripotent stem cells (iPSC), gene-edited to be homozygous for the c.694T>G/ p.(Phe232Val) variant, demonstrated lower EIF3F protein levels and reduced proliferation rates [9]. Furthermore, both heterozygous and homozygous variants reduced translation rates in iPSC cells [9], suggesting a *loss-of-function* mechanism.

In the current study, we assembled a group of 21 previously unreported individuals with homozygosity or compound heterozygosity for the variant c.694T>G/ p.(Phe232Val). We refine the *EIF3F*-related phenotypic spectrum in this group and describe an additional, so far unreported disease-causing variant. Thus, we confirm *EIF3F*-deficiency as a relatively prevalent cause for autosomal-recessive NDD.

## Methods

### Editorial policies and ethical considerations

Individual genetic testing was performed either in diagnostic or research settings. The study was approved by the ethical committee of the Friedrich-Alexander-Universität Erlangen-Nürnberg, and by local institutional review boards of the contributing institutions where applicable.

### Affected individuals

The study group was gathered through GeneMatcher [14], and internal collaborations, such as the corresponding authors of [9]. Parents or the legal guardians gave written informed consent before enrollment. Investigations were conducted according to Declaration of Helsinki principles.

### Methods

A questionnaire filled out by the patient’s clinical team was used to collect detailed clinical characteristics of affected individuals with bi-allelic *EIF3F* variants. To normalize body measurements (height, weight and head circumference) to the corresponding age, the publicly available data-source “https://www.pedz.de/” based on birth data by Voigt et al. [17] and studies reported by Kromeyer-Hauschild et al. [7] for older age groups were used.

*EIF3F* variants were identified by sequencing of autism/ intellectual disability gene panels, whole exome sequencing (WES) or whole genome sequencing, performed as a clinical test or within a research project as described previously [4, 12, 13], or by Sanger sequencing for co-segregation testing in core family members, as presented in Additional file 1: Table S1 and Additional file 2: Fig. S1.

We used genotypes of 136 frequent SNPs (MAF > 5%) generated from internal WES data (1818 independent samples, affected individual and parents of P13 and one affected individual of P14) covering *EIF3F* and its flanking regions (chr11: 7,614,107–8,413,933 (hg19)). We defined haplotype blocks using the definition of the model “solid spine of linkage disequilibrium (LD)” in Haploview [2] and determined haplotypes at an individual basis using PHASE vs.2.1.1 [15] as described previously [5]. Within the LD block of *EIF3F*, five of seven common SNPs (rs79714374, rs12421289, rs12278319, rs7941782, rs4758267, rs12420464, rs56392532) were identified to be tagging SNPs for six different haplotypes with frequencies between 3.5 and 53.4%. The set of seven SNP was used to assess the haplotypes in other affected individuals with the homozygous *EIF3F* missense variant and if available, their parents. Some redundantly tagged SNPs (rs79714374 and rs56392532) had a coverage of < 10 × in affected individuals of P2, P3, P6, P10, P12, P17 (Table 1), but due to very high linkage disequilibrium in 1818 WES, their genotypes could be tagged by rs12420464 and rs12421289, respectively. Genotypes of rs12278319 in mother of P10 and of rs79714374 and rs12420464 in the affected individual of P17 could be inferred in single individuals of P10, P17 due to available genotypes in other core family members, and the ones of two SNPs in P4 (rs79714374, rs12420464) could be deduced to one of the haplotypes identified in 1818 WES data. Due to insufficient coverage of two SNPs (< 15x) in the affected individual of P16, the DNA was sequenced by Sanger for rs12278319 and rs12420464.

**Table 1:** Haplotypes at *EIF3F* in 15 families

Pedigree	Combination of haplotypes in…		
	…Affected individual	…Mother (frequency of 2nd haplotype)	…Father (frequency of 2nd haplotype)
P1	CCACCGC/CCACCGC	CCACCGC/CCACTGC (0.534)	CCACCGC/CTGCTGT (0.124)
P2	[C]CACCGC/[C]CACCGC	[C]CACCGC/[C]CACTGC (0.534)	[C]CACCGC/[C]CGACGC (0.158)
P3	[C]CACCGC/[C]CGACGC^§^	[C]CACCG[C]/[C]CACTG[C] (0.534)	[C]CGACG[C]^§^/[C]CACTG[C] (0.534)
P4	[c]CACC[g]C/[c]CACC[g]C	[c]CACC[g]C/[c]CGCC[c]C (0.095)	[c]CACC[g]C/[c]CACT[g]C (0.534)
P6	CCACCGC/CCACCGC	[C]CACCGC/[C]CGCCGC (0.095)	[C]CACCG[C]/[C]CGACG[C] (0.158)
P7	CCACCGC/CCACCGC	CCACCGC/CTGCTGT (0.124)	CCACCGC/CCACCGC (0.035)
P9	CCACCGC/CCACCGC	CCACCGC/CCACTGC (0.534)	CCACCGC/CCGCCGC (0.095)
P10	[C]CACCGC/[C]CACCGC	[C]CCCG[C]/[C]C[x]CTG[C] (n.a.)	[C]CACCG[C]/[C]CACTG[C] (0.534)
P11	CCACCGC/CCACCGC	CCACCGC/CCACTGC (0.534)	CCACC[G]C/CCACT[G]C (0.534)
P12	[C]CACCGC/[C]CACCGC	CCACCGC/TTGCTTT (0.047)	[C]CACCGC/[C]CACTGC (0.534)
P13	CCACCGC/CCACCGC	CCACCGC/CCGCCGC (0.095)	CCACCGC/CCACTGC (0.534)
P14	CCACCGC/CCACCGC	No WES	No WES
P15	CCACCGC/CCACCGC	No WES	No WES
P16	CCACCGC/CCACCGC	No WES	No WES
P17	{C}CACC{G}C/{C}CACC{G}C	CCACC[G]C/CTGCT[G]T (0.124)	CCACCGC/CCACTGC (0.534)

For haplotype analyses, seven intragenic SNPs were used: rs79714374, rs12421289, rs12278319, rs7941782, rs4758267, rs12420464 and rs56392532. Haplotype C–C-A-C–C-G-C harboring the missense variant, had a frequency of 3.5% in 1818 independent, house-internal control WES from Germany. Genotypes of rs79714374 and rs56392532 in [brackets] had a low coverage, but due to very high linkage disequilibrium, their genotypes in individuals of P2, P3, P6, P10, P12, P17 could be tagged by rs12420464 and rs12421289, respectively. Genotypes in {other brackets} could be inferred in single individuals of P10, P17 due to available genotypes in other core family members and the ones in lowercase in [brackets] in P4 from haplotypes in 1818 control WES

P, pedigree; n.a, not applicable

^§^c.861dup/ p.(Gln288AlafsTer14) is on the underlined haplotype in P3 (frequency of 0.158 in controls)

## Results

**Fig. 1:**
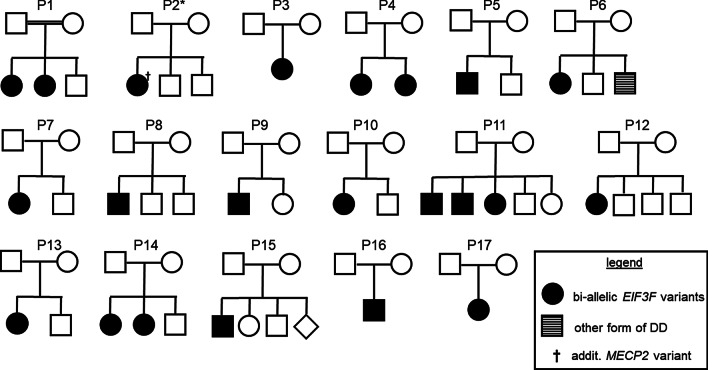
Pedigrees of 17 families with *EIF3F*-related NDD. All parents, but the ones of P16 were identified as heterozygous carriers of *EIF3F* variants. P3 is the family with the compound heterozygous individual. P, pedigree. ^†^Individual with an additional, de novo* MECP2* variant. DD, developmental delay; P, pedigree

We identified the same homozygous missense variant c.694T>G/ p.(Phe232Val) in *EIF3F* (NM_003754) in 21 individuals from 16 families (Fig. 1). All tested parents were heterozygous carriers of the variant (parents of pedigree 16 (P16) were not available for testing). Parental consanguinity was reported in one of 16 families. An additional affected individual (P3) was compound heterozygous for the maternally inherited missense variant c.694T>G/ p.(Phe232Val) and a paternally inherited variant c.861dup/ p.(Gln288AlafsTer14). The latter variant was absent in the Genome Aggregation Database (gnomAD) [6], and was predicted to result in a frameshift and subsequently either in mRNA decay or a truncated protein (length reduced by 20%, exon 6 of 8) with altered structure.

The female individual of family P2 had two molecular diagnoses. In addition to the homozygous *EIF3F* variant, she had a de novo frameshift variant in *MECP2*, implicated in Rett syndrome (OMIM #312750). As symptoms in *EIF3F*-related NDD considerably overlap with those in Rett syndrome, we did not include this individual in the summary of clinical aspects (Table 2), but presented details in Additional file 1: Table S1.

**Table 2:** Clinical features of individuals with bi-allelic *EIF3F* variants

Feature	Current study (total no. of indiv. with data; percent of aff. indiv.)	Published study (total no. of indiv. with data; percent of aff. indiv.) [9]
Homozygous for c.694T>G/ p.(Phe232Val)	16 (17; 94%)	9 (9; 100%)
Parental consanguinity	1 (17; 6%)	0 (7; 0%)
Family history		
Affected sibling(s) (bi-allelic *EIF3F* variants)	4 (17; 24%)	2 (9; 22%)
Parents with neurological symptoms	2 parents (34; 6%; epilepsy or migraines)	1 parent (14; 7%; mild ID)
Gender		
Female	15 (68%)	5 (56%)
Male	7 (32%)	4 (44%)
Average age at last examination in years (median)	12.1 ± 9.6 (8.5)	16.3 ± 13.4 (13.0)
Pregnancy/delivery		
Eventful pregnancy	1 (21; 5%; oligo-hydramnios)	1 (9; 11%; abnormal prenatal scan)
Premature delivery	2 (20; 10%)	0 (9; 0%)
Perinatal asphyxia	1 (19; 5%; suspected)	0 (9; 0%)
Development		
Global developmental delay	21 (21; 100%)	9 (9, 100%)
Speech delay	21 (21; 100%)	n.a
No speech	5 (21; 24%)	n.a
Regression	3 (21; 14%)	n.a
Behavioral problems	12 (21; 57%)	6 (9; 33%)
Hearing loss	12 (21; 57%)	3 (9; 33%)
Muscular hypo-/hypertonia	10 (21; 48%)	(%)
Ophthalmological findings		
Hyper-/myopia	8 (21; 38%)	
Strabismus	3 (21; 14%)	1 (9; 11%)
Nystagmus	1 (21; 5%)	
Coloboma	1 (21; 5%)	
Brain imaging		
Nonspecific findings	5 (13; 38%)	3 (7; 43%)
Sleeping problems	5 (21; 24%)	n.a
Epilepsy—confirmed	3 (20; 15%)	6 (7; 86%)
Other neurological issues		
Encephalopathy	1 (21; 5%)	n.a
Meningioma	2 (21; 10%)	
Psychosis	1 (21; 5%)	
Body measurements		
Microcephaly at birth	4 (10; 40%)	0 (1; 0%)
Short stature at birth	3 (15; 20%)	n.a
Microcephaly later	6 (19; 32%)	1 (8; 13%)
Short stature later	8 (20; 40%)	1 (4; 25%)
Malformations		
Cleft lip/ palate (incl. minor form)	2 (20; 10%)	1 (9; 11%)
Gastrointestinal symptoms	5 (21; 24%)	n.a
Dysmorphisms		
Fine facial features	2 (19; 11%)	n.a
Findings of nose	5 (20; 25%)	
Posteriorly rotated ears	7 (20; 35%)	
Deep set or encased nails of fingers and/ or toes	6 (20; 30%)	n.a
Abnormality 5th finger/ toe (shortness, clinodactyly)	3 (20; 15%)	
Short hands/ feet or slender fingers/ toes	5 (20; 25%)	
Flat feet	3 (20; 15%)	

Each row indicates the number of individuals/ families with the specified feature (number in parantheses indicate number of individuals with available information on this feature and percentage). Due to an additional confounding diagnosis of *MECP2*-related disorder in affected individual of P2 and the issue of overlapping phenotypes, we considered this individual only for the first four categories, but no further aspects

aff. indiv.: affected individuals; incl.: including; n.a. not applicable; No./ no.: number

Among the reported individuals were 15 females (14 total when excluding the individual with Rett syndrome) and 7 males. Overall, history of pregnancies and deliveries were largely uneventful, although oligohydramnios was noted in one pregnancy (P9), and perinatal asphyxia suspected in another case (P14). The average age at the time of the last physical examination was 12.1 ± 9.6 years (mean ± standard deviation), with a median of 8.5 years; four individuals had an age of > 18 years.

All affected individuals had developmental delays. Considering motor milestones, 33% of ascertained individuals (4/12) exhibited delays in unassisted sitting (> 10 months) and 70% of individuals (14/20) in independent walking (≥ 18 months, Additional file 1: Table S1). The single individual with compound heterozygous *EIF3F* variants (P3) did not walk independently at the age of 5 years, but crawled. 24% of individuals (5/21) had not developed speech at the last examination including the compound heterozygous individual, while speech abilities varied widely between few words and simplified, but usable language in the remaining individuals.

Hearing loss was reported in 57% of affected individuals (12/21). More than half of the probands (12/21; 57%) were observed to have behavioral problems such as obsessive compulsory disorder, social problems, anxiety, autism, hyperactivity, attention deficit, aggressivity or pica. Muscular hypo- or hypertonia was also common and diagnosed in 48% of affected individuals (10/21). Notably, different ophthalmological findings were observed in up to 38% of affected individuals (8/21) and included hyper-/myopia (38%), strabismus (14%), nystagmus (5%) and coloboma (5%). Brain imaging revealed nonspecific findings in five of 13 examined affected individuals (38%). Five of 21 individuals (24%) had sleeping problems. Epilepsy was diagnosed in 15% of individuals (3/20).

Regression of cognitive abilities was observed in three of 21 individuals at an age of 2.5 years (P11.1), 21 years (P4.1) and in adulthood (P14). Two of these lost their speech abilities, while the third developed mood swings and demanded more attention. P14 had a combination of meningioma and psychosis, and an additional individual (P16) was operated on a meningioma.

Two individuals were noted to have reduced pain sensitivity. In one proband, muscle atrophy was noted, while in two of the ten individuals with altered muscular tone, a combination of truncal hypotonia and hypertonic extremities was diagnosed.

Short stature was observed in 40% of affected individuals at the last physical examination (Table 2, Additional file 1: Table S1). Interestingly, four of the seven individuals with short stature at the last examination (with available data of body length at birth and at the last examination), had normal body length at birth. Microcephaly was common at the last physical examination (6/19; 32%), and microcephaly (4/10; 40%) or normocephaly (6/10; 60%) remained consistent between birth and the last measurement. Notably, the compound heterozygous individual of P3 was both short for age and microcephalic.

**Fig. 2:**
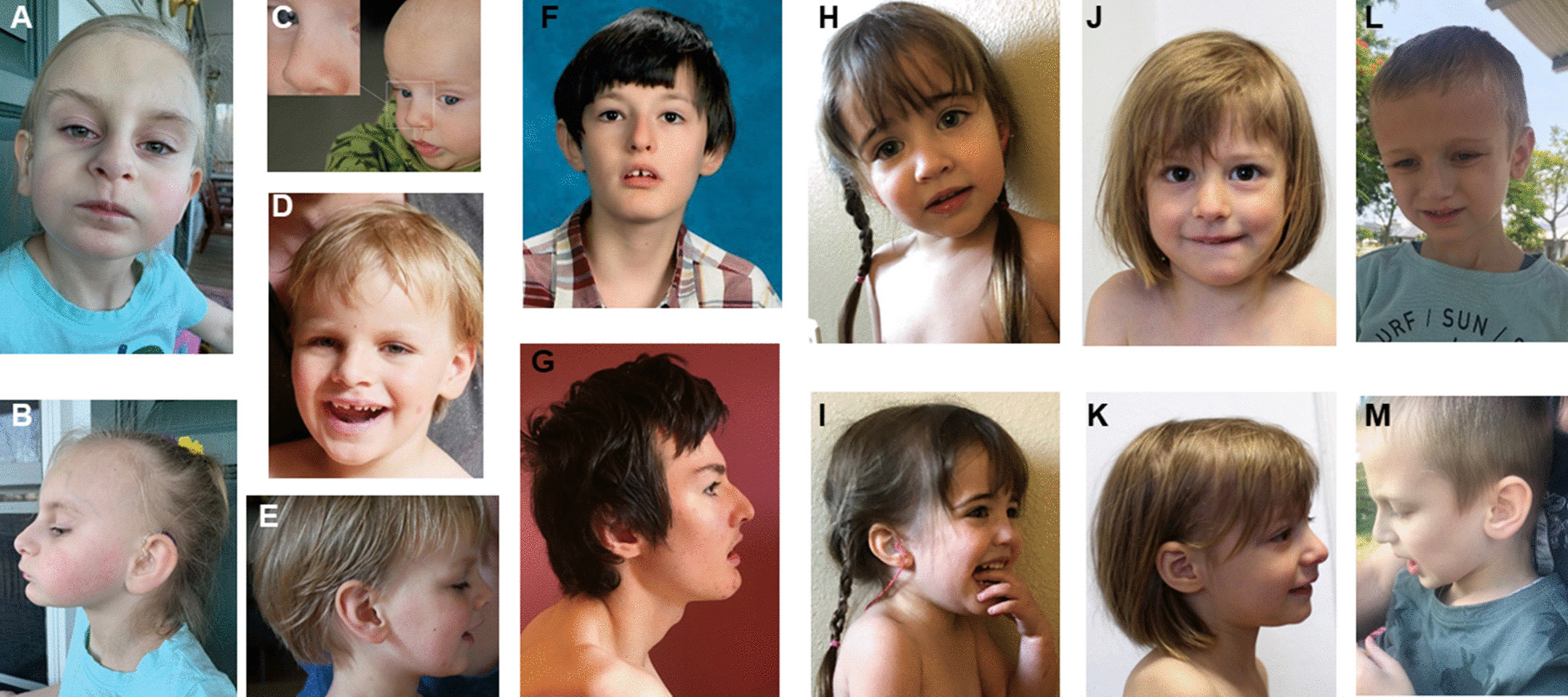
Frontal and lateral facial profiles of six affected individuals carrying bi-allelic *EIF3F* variants. **a**, **b** represent P3 at 5y, **c** P5 at 4m and **d**, **e** P5 at 4y 3m, **f**, **g** show P9 at 13y and at 17y, respectively, **h**, **i** P12 at 2y 8m, **j**, **k** P13 at 3y 2m and **l**, **m** P15 at 6y 8m. All affected individuals have fairly even palpebral fissures, a pointed nasal tip which is rather prominent for age at lateral view. Note nasal fistula and groove at left lip in P5 before surgery (**c**), and after correction (**d**). *y = years*;* m = months*

Five of the individuals (24%) had gastrointestinal problems which included gastro-esophageal reflux disease, difficulties to swallow, alternating constipation and diarrhea and neonatal feeding problems. Cleft lip and palate (P15), tetralogy of Fallot (P3) or combination (P5) of a groove of lip (considered a minor variant of cleft lip, Fig. 2c, d) and a nasal fistula (Fig. 2c) were observed in a single individual each. The compound heterozygous individual had gastrointestinal problems and a congenital heart defect.

More prevalent dysmorphic findings included a tubular and/ or narrow nose, a pointed nasal tip and anteverted nares. We also observed posteriorly rotated ears, short and/ or encased (= embedded by abundant skin) finger and toe nails (Fig. 2, Table 2). Also, palpebral fissures were fairly even in all affected individuals (Fig. 2). A unilateral single palmar crease was noted in four individuals. Dysmorphic features were generally subtle and non-specific and were not considered a recognizable facial gestalt.

Two individuals had initially received targeted diagnostics for short stature or failure to thrive including a gene panel for short stature and testing for Russel-Silver syndrome (OMIM #180860). The individual compound heterozygous for *EIF3F* variants presented with intrauterine growth retardation. Most other genetic testing that was performed in affected individuals (Angelman syndrome, Fragile X-syndrome) overlapped those reported previously [9].

Heterozygous carriers were mainly asymptomatic. One father was reported to have epilepsy, and one mother migraines. Those overlapping symptoms are likely unrelated and of different etiology.

To test whether the missense variant arose once or recurrently, we performed haplotype analyses. This revealed that the *EIF3F* variant was on an identical haplotype (minimal 7.8 kb) in 15 affected individuals of all tested families, suggesting a founder variant (Table 1).

## Discussion

Our study confirms a relevant role of *EIF3F* in syndromic NDD. We observed the same pathogenic homozygous missense variant c.694T>G/ p.(Phe232Val) in all but one affected individual. This variant represents the 7^th^ most common *EIF3F* missense variant in gnomAD (0.07%) with highest frequencies in Ashkenazi Jewish (0.21%) and non-Finnish European individuals (0.12%) [6]. In line with its pathogenicity, no individual in gnomAD was reported to be homozygous for this variant, in contrast to five of the six more frequent variants. The haplotype analyses performed in this study add the finding that the variant most probably arose once on a single haplotype, indicating a founder variant.

*EIF3F* was previously reported to be one of few genes significantly enriched for bi-allelic genotypes in large cohorts of individuals with NDD, due to the shared variant c.694T>G/ p.(Phe232Val) in all affected individuals [9]. The frequency of heterozygous carriers in gnomAD, especially in Non-Finnish European and Askenazi Jewish individuals, is higher than for most other autosomal recessive NDDs. For many of the other autosomal recessive NDDs, only few or a handful of families have been reported [1, 11]. In those diseases, predominantly truncating variants are causative. Therefore, an identical, rather frequent missense variant in *EIF3F* in almost all affected individuals is an uncommon finding in NDDs, particularly in a cohort with heterogeneous, ethnical and regional backgrounds. Particularly common pathogenic variants have been observed in certain other genetic diseases: e.g. p.Phe508del variant in *CFTR* in cystic fibrosis in Europeans, population-specific variants in *HFE* in hemochromatosis, and population-specific truncating variants in *GJB2* in deafness. In this study, the *EIF3F* missense variant was identified in affected individuals of a wide spectrum of European/ West Asian origins including French, English, Irish, Scottish, German, Bulgarian, Ukranian, Russian, Ashkenazi Jewish, and Iraqi. Haplotype analyses support a single mutational event on a founder haplotype, while the nascence of the missense variant cannot be determined to a more localized region.

As only the identical missense variant in *EIF3F* has been functionally characterized and shown to result in reduced protein amount/ stability, decreased proliferation rate and reduced translational rate, it remains speculative whether other missense variants might have comparable effects. Other rare missense variants might be functionally irrelevant or less harmful, as they might reach a certain harmful threshold causing NDD as in case of c.694T>G/ p.(Phe232Val) [9]. In contrast, the finding of the rather severe phenotype in the individual with compound heterozygous variants in *EIF3F* suggests some genotype–phenotype correlation and a possibly residual EIF3F function in individuals homozygous for c.694T>G/ p.(Phe232Val). Of note, the overall number of truncating alleles in individuals in gnomAD v2.1.1 is extremely low (probability of being *loss-of-function* intolerant = 0.97, observed over expected variants = 0.07). Regarding lack of individuals with two truncating variants and of further affected individuals who are compound heterozygous for truncating variants, one might speculate that truncating variants on both alleles might not be compatible with life.

In this study, all affected individuals had global developmental delay of variable degree. Motor developmental delay was variable: one patient did not learn to walk independently, while most individuals learned walking late, and some individuals achieved motor milestones at a normal age. Similarly, the degree of ID varied widely. This study adds the finding of significant speech delay to the phenotype: the majority of individuals spoke more than few words, while a quarter of affected individuals had no speech development. In comparison to the initial report [9], hearing loss and behavioral difficulties were more common findings, and epilepsy less frequent in this study. Other frequent, so far unreported symptoms observed in this study are muscular hypotonia and/ or hypertonia, ophthalmologic findings and sleeping problems. Results of a murine study might indicate some concordance with the human phenotype and support *loss-of-function* as the underlying cause: partial depletion of murine eIF3f amplified muscle atrophy compared to wild-type mice and reduced the MTOR pathway activation [3]. In regard to this study’s ophthalmologic findings, some of these (hyper-/ myopia) might not necessarily be related to EIF3F deficiency, as they are common in the general population.

**Fig. 3:**
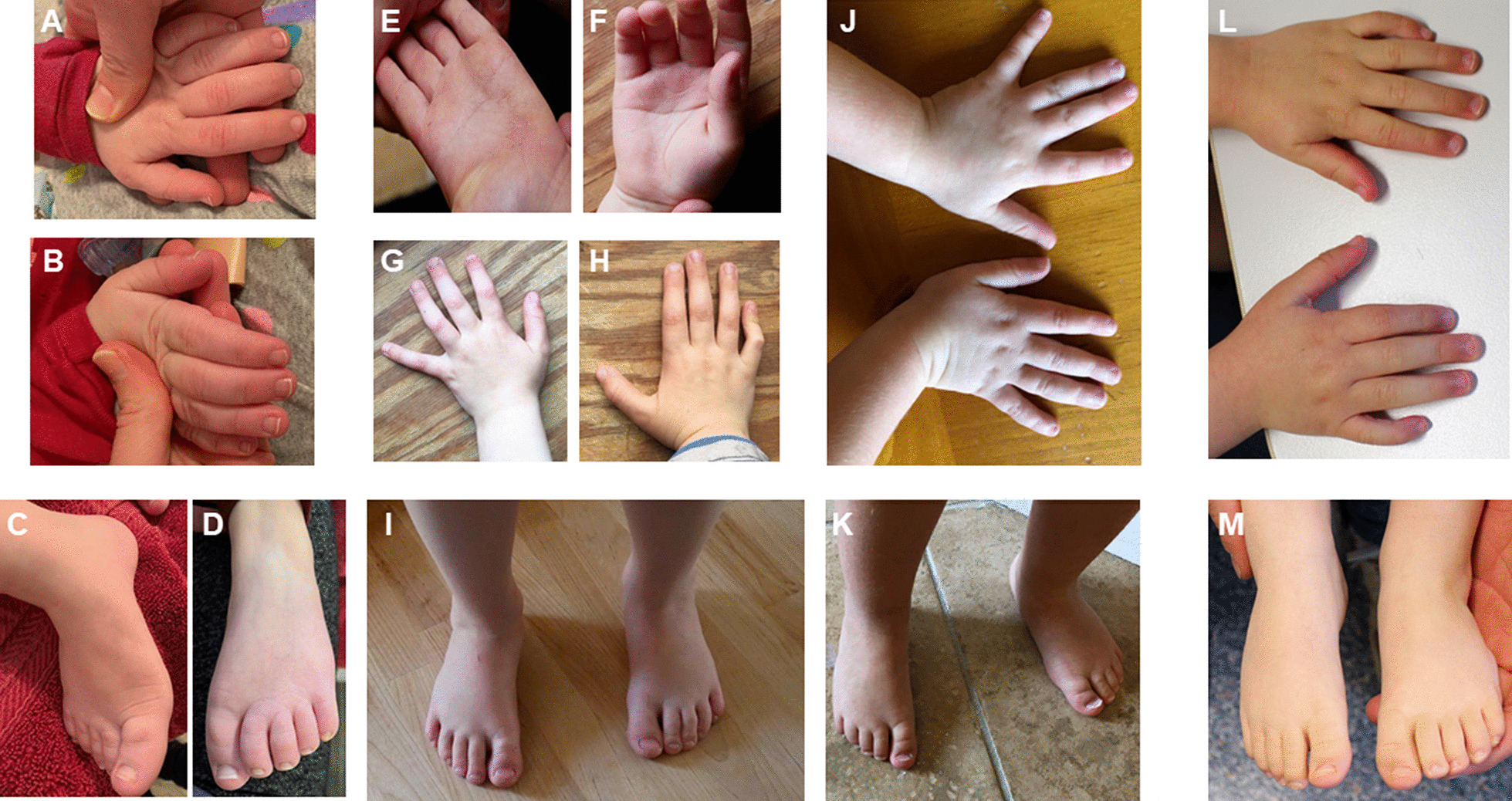
Hands and feet of four affected individuals carrying bi-allelic *EIF3F* variants. **a**–**d** represent P3 at 5y, **e**–**i** P5 at 4y 3m, **j**, **k** P12 at 2y 8m, and **l**, **m** P13 at 3y 2m. Some affected individuals have puffy backs of hands and feet; most individuals have encased nails (= nails embedded by skin), predominantly of the finger nails and short toe nails. *y = years*;* m = months*

The observation of reduced pain sensitivity in this study supports an association with this previously described, however rare symptom [9], as did the finding of muscular atrophy/ muscular hypoplasia in another individual. In concordance with the previous study, brain imaging did not reveal specific findings and were therefore not considered diagnostically indicative in *EIF3F* related NDD. Thus, genome-wide sequencing approaches (genome or exome sequencing) represent an essential component of the diagnostic work-up.

Developmental regression or neurodegeneration at various ages (2.5 to ~ 30 years) was observed in three of the 20 affected individuals which might be relevant for prognosis. However, two of the three individuals had additional diagnoses that are not necessarily related to this syndromic disorder: encephalopathy in an individual with vitamin B12 deficiency and psychosis in an individual with meningioma. In another individual, an increased seizure frequency also led to the diagnosis of meningioma. The cohort size and the relatively young ages of the majority of individuals did not allow conclusions as to whether those symptoms are part of the disease spectrum or might have an independent cause. In large collections of malignancies (COSMIC; accessed on 3^rd^ of December 2020 at cancer.sanger.ac.uk), somatic *EIF3F* variants have been detected in 0.9% of 38,579 samples (n = 353) and did not include 130 meningioma samples, providing no further evidence for a role of EIF3F in tumorigenesis of meningioma [16].

Short stature, also occurring until adulthood, was commonly observed in this study group. Microcephaly was less frequent than short stature and of variable degree within this cohort, while longitudinal data indicated that head growth had a more constant course along the centiles than height.

Rare features that might be part of the *EIF3F* related NDD include functional problems of the gastrointestinal tract, undescended testes, cleft lip and palate, heart defect and nasal fistula. Of those, the latter two had not been previously described. In agreement with the previous study, we did not recognize an obvious distinctive facial appearance. While the previous study had indicated tapered fingers and dysplastic toe nails [9], we observed nasal findings (tubular nose, pointed nasal tip, anteverted nares), posteriorly rotated ears as well as short and/ or encased finger and toe nails as more frequent, recurrent features.

In summary, this study confirms the previously reported *EIF3F* missense variant as a relatively frequent cause of autosomal-recessive NDD. Characteristic features include global developmental delay, delayed speech development, behavioral difficulties, altered muscular tone, hearing loss, ophthalmological symptoms, short stature, and minor anomalies of the ears, nose, hands and feet.

## Supplementary Information

**Additional file 1: Table S1**. Detailed clinical information.

**Additional file 2: Fig S1**. Examples of sequences of disease-causing variant c.694T>G/ p.(Phe232Val) at individual basis.

## Data Availability

All data generated or analyzed during this study are included in this published article and its supplementary information files.
