# Supplementary figures and images for: EIF3F-related neurodevelopmental disorder: refining the phenotypic and expanding the molecular spectrum
Source: Orphanet J Rare Dis. 2021 Mar 18;16:136. doi: 10.1186/s13023-021-01744-1 (PMC7977188; doi:10.1186/s13023-021-01744-1)

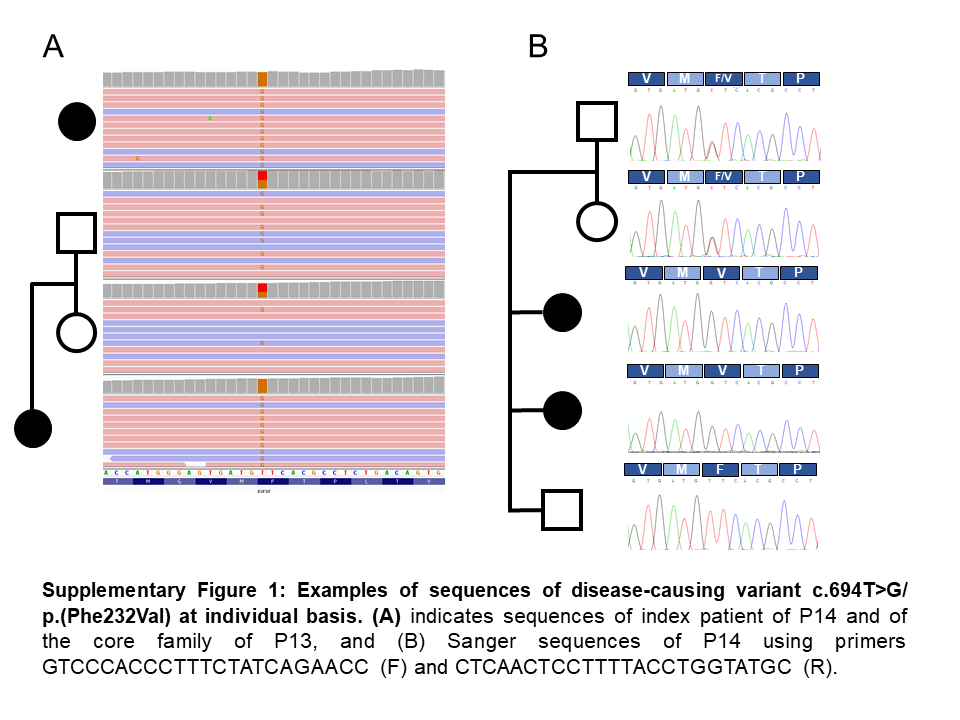

Supplement: Supplementary file 2 — Additional file 2: Fig S1. Examples of sequences of disease-causing variant c.694T>G/ p.(Phe232Val) at individual basis. [file 13023_2021_1744_MOESM2_ESM.tif]
